# Design and Synthesis of Ag Nanocluster Molecular Beacon for Adenosine Triphosphate Detection

**DOI:** 10.1155/2019/2786156

**Published:** 2019-10-13

**Authors:** Xiaoshuang Li, Hao Zhang, Ying Zhao, Lili Lian, Xiyue Wang, Wenxiu Gao, Bo Zhu, Dawei Lou

**Affiliations:** Department of Analytical Chemistry, Jilin Institute of Chemical Technology, 45 Chengde Street, Jilin 132022, China

## Abstract

This study presents a fluorescence method for detecting adenosine triphosphate (ATP) based on a label-free Ag nanocluster molecular beacon (MB) with high sensitivity. The sensor contains a hairpin-shaped MB, two short single-stranded DNA strands, and T4 DNA ligase. The MB consists of three parts, which are the template DNA sequence for synthesizing Ag nanoclusters at the 5′ end, the middle DNA with a hairpin-shaped structure, and the guanine base-rich DNA sequence at the 3′ end. The sensor exhibits high fluorescence intensity in the absence of ATP. However, when the probe is used for ATP detection, the two short DNA sequences in the sensor would form a long sequence by enzymatic ligation reaction; this long sequence opens the hairpin-shaped structure of the MB and decreases the fluorescence of the system. Under optimal analytical conditions, a clear linear relationship is observed between ATP concentration and fluorescence intensity in the range of 0.1–10 *μ*M. The interference presented by other small molecules during ATP detection is evaluated, and results confirm the good selectivity of the proposed sensor. Compared with traditional methods, the sensor is label free, easy to operate, inexpensive, and highly sensitive.

## 1. Introduction

Adenosine triphosphate (ATP) is the energy source of cells; it stores and transmits chemical energy in the life system. ATP can regulate various chemical reactions in organisms and metabolic processes in cells [1]. The molecule is an unstable high-energy compound consisting of one molecule of adenine, one ribose, and three molecular phosphate groups. ATP exists in various organisms, and the amount of ATP available directly affects the health of an organism [2]. Too high or too low ATP concentrations can lead to various diseases, such as hypoglycemia [3], Parkinson's disease [4], cardiovascular diseases, and Fe deficiency. Besides its role in energy metabolism, ATP completes the replication and transcription of DNA. ATP is also used as an indicator to detect cell viability and damage in biological cells [5]. Therefore, ATP concentrations should be detected.

The traditional methods of ATP detection include fluorescence, electrochemistry [6], colorimetry, chromatography, and bioluminescence. These methods, however, present several limitations in ATP detection. For example, chromatographic detection requires long reaction times. Bioluminescence requires the connection of enzymes, which is complicated and expensive [7]. The detection limit of the aptamer method is relatively high. Therefore, a fast, low-cost, and ultra-sensitive method for ATP detection should be developed.

Molecular beacon (MB) is fluorogenic oligonucleotide probes with a hairpin-shaped structure used for detecting specific nucleic acid sequences. The design of MBs is ingenious. MBs have a short chain with a hairpin-shaped structure and are fluorescently labeled on oligonucleotide chains [8]. MBs are generally divided into two parts, namely, a ring area and a stem area. The circular region can specifically bind to the target molecule, and the fluorescein and quenching groups are connected to the two ends of the stem region. In the absence of the target molecule, the fluorophore and quenching groups are in close proximity and the fluorescence is quenched. The spatial structure of the MB changes when the target molecule is bound, and the fluorophore and the quenching group are separated, resulting in fluorescence recovery [9, 10]. Tyagi designed and invented MBs in 1996 and used them for analysis of DNA/RNA hybridization in living cells [11], real-time monitoring of the polymerase chain reaction [12], and quantitative detection of DNA/RNA [13, 14]. However, the stability of MBs is poor and cannot be used for clinical applications [15], the labeling cost of the fluorescent and quenching groups at the two ends is expensive, and the required reaction time is long [16]. Therefore, a label-free MB should be developed. Label-free MB fluorescence detection indicates that the ends of MBs need not be marked. Compared with traditional MBs, label-free MBs have simple operation, high sensitivity, stable structure, low cost, and low biological toxicity [17], as such; they have attracted considerable attention in recent years [18].

Fluorescent nanomaterials present unique electrical, optical, magnetic, and thermal properties and have been widely applied to various industries. Common fluorescent nanomaterials include metallic fluorescent nanoclusters [19], graphene [20], and quantum dots [21]. Commonly used metal fluorescent nanoclusters (Ag, gold, and copper nanoclusters) are widely used due to their stable fluorescence properties, high fluorescence intensity, and good biocompatibility. The most common method used for synthesizing DNA-Ag nanoclusters is through a DNA template. DNA nanoclusters are usually synthesized from zeolitic molecular sieves, polymers, small molecules, proteins, and DNA as templates. DNA as templates is simple and easy to obtain, and the generated DNA-Ag nanoclusters have stable fluorescence properties and good biocompatibility. The base sequence lengths of the template and its arrangement have different effects on DNA-Ag nanoclusters when DNA is used as a template for synthesis [22]. Ag^+^ strongly interacts with DNA bases during preparation. NaBH_4_ as a reducing agent can reduce metal atoms into metal clusters [23]. During preparation of DNA-Ag nanoclusters, DNA sequences rich in guanine bases (G) were observed to approach the Ag nanoclusters after hybridization of DNA sequences rich in G with DNA-Ag nanoclusters; the fluorescence emission intensity is approximately several hundred times stronger than the original value [24]. DNA-Ag nanoclusters, such as Hg^+^, Pd^2+^, and Cu^2+^, and small molecules, such as DNA, RNA, and glucose, can be used as high-sensitivity detection ions for fluorescent probes, cell imaging [25, 26], and fluorescence immunoassay testing [27, 28].

In this study, we present a fluorescence method for ATP detection based on a label-free Ag nanocluster MB with low cost and high sensitivity. The label-free MB is synthesized without fluorescent dye labeling and DNA terminal modification. Ag nanoclusters are formed at the 5′ ends of the MB, and the hairpin-shaped structure of the MB makes the 3′ end G-rich sequence close to the Ag nanoclusters, resulting in a fluorescence enhancement. However, the sensor shows an obvious fluorescence decrease with two short DNA strands and T4 DNA ligase in the presence of ATP. T4 DNA ligase plays a key role in the sensor [29]. The ligase is one of the most commonly used enzymes for various biotechnological researches and plays a pivotal role in the ATP-dependent DNA replication. They catalyze the formation of a phosphodiester bond between juxtaposed 3′ hydroxy and 5′ phosphate termini in double-stranded DNA [30]. And, ATP is the cofactor of the T4 DNA ligase with specific dependence in the enzymatic ligation reaction. Hence, the hairpin-shaped MB would transform to a stable double helix strand in the presence of ATP, two short DNA strands, and T4 DNA ligase. Then, the 5′ and 3′ ends are spatially separated, resulting in a decrease in fluorescence. Hence, the ATP concentration can be detected from the decrease in fluorescence intensity of the sensor. Compared with traditional methods, the proposed sensor is label free, easy to operate, inexpensive, and highly sensitive.

## 2. Experiments

### 2.1. Apparatus

Fluorescence measurement was conducted by using a Shimadzu RF-5301 PC spectrophotometer. The particle sizes of the Ag nanoclusters were characterized with transmission electron microscopy (TEM) using an FEI Tecnai G^2^ F20 S-TWIN instrument operated at 200 kV. The excitation wavelength of the fluorescence spectrum was fixed to 570 nm. SHA-C reciprocating water bath oscillators were also used in the experiment. The pH meter used in the experiment was a Sartorius PB-10 pH meter (Sartorius Instruments Co. Ltd., Beijing, China). Ultra-pure water was obtained from Milli-Q Reagent Water System (Millipore, Bedford, MA, USA).

### 2.2. Reagent

The DNA oligonucleotides used for the synthesis of Ag nanoclusters in the experiment were purchased from Shenggong Biotechnology Co. Ltd. (Shanghai, China). The DNA oligonucleotide sequences are as follows:

  Molecular beacon (MB): 5′-CCC TTA ATC CCC GTT GAC GAG TCT AGT TCT TGT CGT CAA CGG GTG GGG TGG GGT GGG G-3′
  DNA c1: 5′-GAC GAC AAG AA-3′
  DNA c2: 5′-PO4-CTA GAC TCG TC-3′
  Complementary DNA (cDNA): 5′-GAC GAC AAG AAC TAG ACT CGT C-3′

AgNO_3_, NaBH_4,_ and MgSO_4_ were purchased from the National Pharmaceutical Group Chemical Reagent Co. Ltd. T4 DNA ligase, ATP, and hydroxymethyl aminomethane buffer (Tris) were purchased from BBI Life Sciences (Shanghai, China). Uridine triphosphate (UTP), cytidine triphosphate (CTP), guanosine triphosphate (GTP), adenosine diphosphate (ADP), adenosine monophosphate (AMP), and bovine serum were also purchased from Shenggong Biotechnology Co. Ltd. (Shanghai, China). Tris-HNO_3_ buffer and MgSO_4_ were dissolved in 1 L of ultra-pure water to obtain a 100 mM Tris-HNO_3_ buffer solution with 20 mM Mg^2+^.

### 2.3. Fluorescence Assay

Firstly, 10 *μ*L of 30 *μ*mol·L^−1^ DNA strands (MB) and 50 *μ*L of 100 mmol·L^−1^ Tris-HNO_3_ buffer (pH 7.4) solution were mixed and reacted for 10 min at 25°C. Next, 10 *μ*L of 30 *μ*mol·L^−1^ DNA strands (DNA c1) and 10 *μ*L of 30 *μ*mol·L^−1^ DNA strands (DNA c2) were added to the mixed solution for 10 min at 25°C. Then, 10 U T4 DNA ligase was added to the mixed solution, which was subsequently incubated for 1 min at 37°C. Finally, 10 *μ*L of different concentrations of ATP was added to the mixed solution, which was incubated for 30 min at 37°C. A mixed solution containing 100 *μ*L of 100 mmol·L^−1^ Tris-HNO_3_ buffer (pH 7.4) was reacted for 10 min at 25°C to synthesize the DNA-Ag nanoclusters. AgNO_3_ (10 *μ*L and 180 *μ*mol·L^−1^) and NaBH_4_ (10 *μ*L and 180 *μ*mol·L^−1^) were added to the mixed solution, and final concentrations of 1 *μ*mol·L^−1^ MB, 6 *μ*mol·L^−1^ AgNO_3_, and 6 *μ*mol·L^−1^ NaBH_4_ were obtained. The 300 *μ*L mixed solution was placed in a water bath and shaken for 6 h at 25°C to form the fluorescent DNA-Ag nanoclusters. All experiments were repeated three times.

## 3. Results and Discussion

### 3.1. Detection Principle

We designed a fluorescence method for ATP detection based on a label-free Ag nanocluster MB with high sensitivity. The MB is a single-stranded oligonucleotide probe containing a template DNA sequence for synthesizing Ag nanoclusters at the 5′ end, a middle DNA sequence with a hairpin-shaped structure, and a G-rich DNA sequence at the 3′ end. Based on the template of the DNA sequence at the 5′ ends, the formed Ag nanoclusters show a weak fluorescence signal by addition of Ag ions and NaBH_4_. Then, a hairpin-shaped structure is formed at the middle of the MB, which causes the G-rich sequence at the 3′ end to approach the Ag nanoclusters at the 5′ ends, and the fluorescence of the Ag nanoclusters would be enhanced [31].

**Figure 1:**
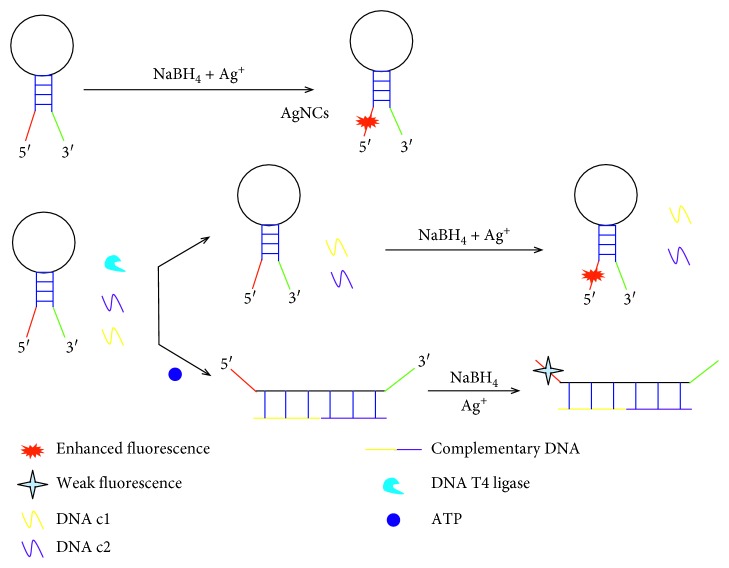
Schematic of ATP detection using a label-free Ag nanocluster molecular beacon.

As shown in Figure 1, the MB maintains a hairpin-shaped structure with the addition of DNA c1, DNA c2, and T4 DNA ligase. However, in the presence of cofactor ATP, the DNA c1 and DNA c2 in the sensor would form a longer sequence as cDNA by the enzymatic ligation reaction. The cDNA provides a complementary DNA base sequence for the middle of the MB and forms a double helix structure with the MB, resulting in the opening of the hairpin-shaped MB. In this case, the 3′-ends Ag nanoclusters and the 5′-ends G-rich DNA bases of the MB are separated, which lead to the disappearance of Ag nanocluster fluorescence enhancement. Hence, we could detect ATP by this sensor because the fluorescence signal decreases directly depends on the concentration of ATP.

### 3.2. Feasibility Analysis

**Figure 2:**
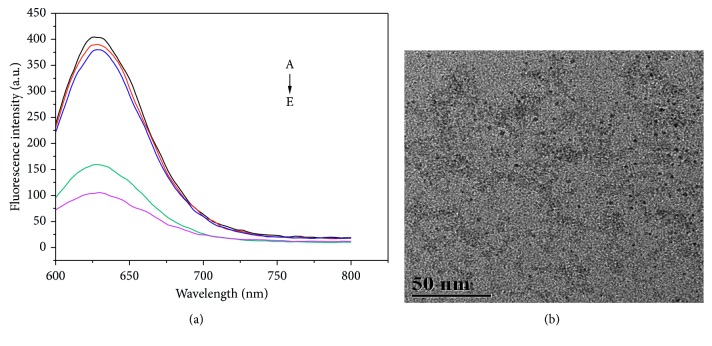
(a) Fluorescence spectra of MB-Ag nanoclusters obtained under different conditions: (A) MB only, (B) MB and ATP, (C) MB, DNA c1, DNA c2, and T4 DNA ligase, (D) MB, DNA c1, DNA c2, T4 DNA ligase, and ATP, and (E) MB and complementary cDNA. (b) The TEM image of the AgNCs.

To verify the feasibility of the label-free fluorescence method for ATP detection, the fluorescence changes of the sensor were investigated under different experimental conditions as follows: (a) MB only, (b) MB and ATP, (c) MB, DNA c1, DNA c2, and T4 DNA ligase, (d) MB and ATP and two short-stranded DNA sequences, and (e) MB and cDNA. The changes of fluorescence intensity are shown (Figure 2(a)). Curve (a) shows that Ag nanoclusters could be synthesized on the template of designed MB and has strong fluorescence intensity with the fluorescence enhancement. As shown in the TEM photo (Figure 2(b)), the Ag nanoclusters are monodispersed, and their particle diameters are 2 to 3 nm. Curve (b) reveals that the addition of ATP does not lead to obvious fluorescence changes in the system. Curve (c) shows that the fluorescence of the system shows small decrease with addition of DNA c1, DNA c2, and T4 DNA ligase, which indicate that the two short DNA strands cannot open the hairpin structure of the MB and T4 DNA ligase cannot work without ATP. Curve (e) indicates that the fluorescence of the system sharply decreases with the addition of a complementary DNA sequence to the middle part of the MB. It is because that cDNA and MB would form a stable double helix structure, resulting in the opening of the hairpin-shaped MB and the separation of the Ag nanoclusters and the G-rich sequence. Due to the disappearance of fluorescence enhancement, the fluorescence intensity is decreased. Finally, Curve (d) reveals that when MB, DNA c1, DNA c2, T4 DNA ligase, and ATP simultaneously exist in the system, the fluorescence of the system would decrease. The introduction of ATP would activate the T4 DNA ligase, and then enzymatic ligation reaction takes place. T4 DNA ligases seal the 5′ PO_4_ and 3′ OH polynucleotide ends, and the two short DNA strands would become a longer-stranded DNA. The long DNA strands can open the hairpin-shaped structure of the MB and separate the 3′ and 5′ ends, resulting in disappearance of fluorescence enhancement. Therefore, the concentrations of ATP could be detected through the decreases of fluorescence intensity in this sensor.

### 3.3. Optimization of Experimental Conditions

To obtain the best experimental conditions of this biosensor for ATP detection, the key experimental factors were optimized.

**Figure 3:**
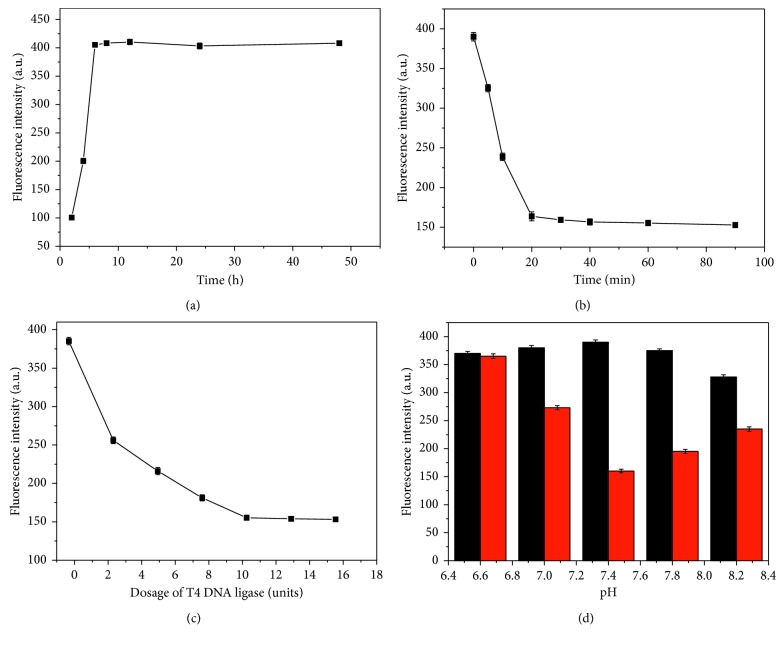
(a) Synthesis time of AgNCs in 2, 4, 6, 8, 12, 24, and 48 hours. (b) Effect of T4 DNA ligase reaction time on fluorescence intensity. The reaction times are 0, 5, 10, 20, 30, 40, 60, and 90 min. (c) Effect of T4 DNA ligase dosage on fluorescence intensity. Ligase dosage of 0, 2.5, 5, 7.5, 10, 12.5, and 15 U. (d) Effect of pH values on fluorescence intensity. pH values of 6.6, 7.0, 7.4, 7.8, and 8.2.

The stability of silver nanoclusters was investigated. We explored the synthesis time of silver nanoclusters in 2, 4, 6, 8, 12, 24, and 48 hours with the MB only (Figure 3(a)). The fluorescence intensity increases significantly with the increase of reaction time. When the reaction time exceeds 6 hours, the fluorescence intensity has almost no changes, which indicate that the silver nanoclusters are stable. Hence, we chose the 6 hours for the silver nanocluster synthesis.

The reaction time of T4 DNA ligase exerts a remarkable effect on fluorescence intensity. Thus, the reaction time of the T4 DNA ligase was optimized using 10 U T4 DNA ligase and 10 *μ*M ATP for 5, 10, 20, 30, 40, 60, and 90 min (Figure 3(b)). The fluorescence intensity rapidly decreases with increasing reaction time from 0 to 20 min, which indicate the enzymatic ligation reaction took place. Then, the trend of fluorescence intensity is not obviously changed when the reaction time exceeds 30 min. It is demonstrated that the enzymatic ligation reaction of DNA c1 and DNA c2 mostly reach destination. Thus, we used 30 min as the reaction time.

The T4 DNA ligase dosage also has an important influence on the sensor. The T4 DNA ligase dosage was optimized with 10 *μ*mol·L^−1^ ATP for 30 min (Figure 3(c)). Ligase dosage of 0, 2.5, 5, 7.5, 10, 12.5, and 15 U were used for optimization. The fluorescence intensity gradually decreased with increasing T4 DNA ligase dosage from 0 to 10 U, and the changes of fluorescence intensity become slow after adding T4 DNA ligase greater than 10 U. The more the ligase molecules, the better the ligation effects in the reaction. Unfortunately, addition of more ligase also increases the cost of the experiment. Thus, we used 10 U T4 DNA ligase for subsequent reactions.

The effects of pH value of buffer solution on the sensor have been investigated. We chose pH values of 6.6, 7.0, 7.4, 7.8, and 8.2 with the presence and absence of ATP (Figure 3(d)). It can be seen that the fluorescence intensity and S/N (signal to noise ratio) reach the highest when the pH value of the buffer solution was 7.4. So we chose the pH value of the buffer solution to be 7.4.

### 3.4. ATP Concentration Detection

**Figure 4:**
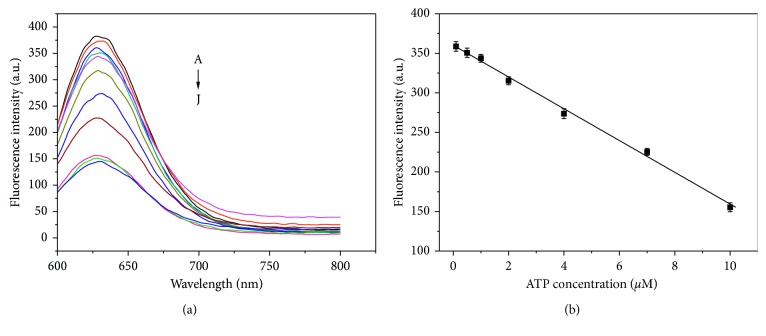
(a) Fluorescence spectra of MB-Ag nanoclusters for ATP detection. The ATP concentrations are (from A to J) 0, 0.01, 0.1, 0.5, 1, 4, 7, 10, 30, and 50 *μ*mol·L^−1^. (b) Linear relationship between ATP concentration and fluorescence intensity. Conditions: 1 *μ*mol·L^−1^ DNA; 6 *μ*mol·L^−1^ Ag; 6 *μ*mol·L^−1^ NaBH_4_; 10 U T4 DNA ligase; and 50 mmol·L^−1^ Tris buffer (pH 7.4).

Under the optimal conditions, the ATP concentration can be detected by the decrease of fluorescence in the system. Samples containing different ATP concentrations were determined, and the results were shown (Figure 4(a)). Fluorescence intensity at 630 nm gradually decreased with the increasing ATP concentration in the range of 0.01–50 *μ*M. This finding is attributed to the fact that the ATP could activate the enzymatic ligation reaction as the cofactor of T4 DNA ligase, which results in separation of the 3′ and 5′ ends of the MB and decrease the fluorescence of the system (Figure 4(b)). The fluorescence intensity shows a clear linear relationship from 0.1 to 10 *μ*M. The linear regression equation is *F* = 360.19–20.08C_ATP_, and the linear correlation coefficient is *R*^2^ = 0.995. The detection limit for ATP is 0.01 *μ*M. The repeatability of the fluorescent biosensor was also measured. The fluorescence biosensor is repeatedly detected with 1 *μ*M ATP, and the relative standard deviation is 3.7% (*n* = 8). Finally, Table 1 shows that the proposed sensor is better than previously reported methods.

### 3.5. Selectivity and Interference

**Figure 5:**
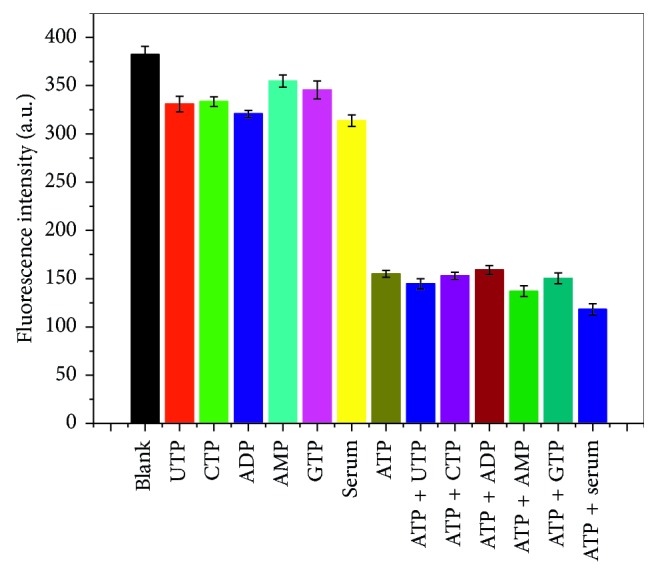
Selectivity and interference of the MB-Ag nanocluster sensing system in ATP detection. ATP is replaced with 10 *μ*M small molecules (e.g., UTP, CTP, ADP, AMP, and GTP) in the detection.

The target sample should be effectively distinguished from other substances. We replaced ATP with 10 *μ*M other small molecules (e.g., UTP, CTP, ADP, AMP, and GTP) to verify the selectivity of the proposed sensor for ATP detection. The experimental procedure and detection method were identical to the ATP detection method (Figure 5). Under the same conditions, only the 10 *μ*M ATP caused the remarkably decrease of fluorescence intensity in the sensor and no remarkable decrease was observed after addition of other small molecules. These results demonstrate that the sensor is selective for ATP detection.

The interferences of the small molecules (UTP, CTP, ADP, AMP, and GTP) were also studied (Figure 5). We combined 10 *μ*M ATP with other small molecules and found no remarkable effect on the fluorescence intensity of the biosensor. This result indicates that other small molecules cannot affect the ATP detection capability of the fluorescent biosensor.

### 3.6. Actual Sample Test

In order to verify the feasibility of ATP detection in practical samples by the sensor, we used bovine serum to simulate real samples for detecting ATP. The results are shown in Table 2. The results showed that the recovery for detecting ATP in bovine serum was 95.8%–103.6% and RSD was less than 2.6%. The results show that ATP can be detected in bovine serum by this molecular beacon silver nanocluster label-free sensing system.

## 4. Conclusions

This study provides a fluorescence method for ATP detection based on a label-free Ag nanocluster MB with high sensitivity and selectivity. Ag nanoclusters are formed at the 5′ ends of the MB, and the fluorescence signal is enhanced in the absence of ATP. However, with the addition of ATP as the cofactor for T4 DNA ligase, the two short DNA sequences in the sensor would provide complementary bases for the middle DNA sequence of the MB to form a double helix structure by the enzymatic ligation reaction, resulting in the opening of the hairpin-shaped MB and the decrease of fluorescence intensity in the sensor. Thus, the ATP concentrations can be detected through the decrease of fluorescence intensity in the sensor. Compared to the traditional methods, this method is label free, inexpensive, and easy to operate and has high sensitivity and selectivity for ATP detection.

## Figures and Tables

**Table 1:** Different methods used for ATP determination.

Serial number	Method	Detection range	Detection limit	References
1	Fluorometric method	0–10 mM	1 mM	[32]
2	Fluorometric method	0.5 *μ*M–1 mM	0.5 *μ*M	[33]
4	Fluorometric method	0.5–17.5 *μ*M	140 nM	[34]
5	Fluorometric method	0.3–20 *μ*M	0.3 *μ*M	[35]
6	Fluorometric method	2–16 *μ*M	1.70 *μ*M	[36]
7	Fluorometric method	5–50 *μ*M	2.07 *μ*M	[37]
8	Colorimetric method	0–0.2 mM	20 *μ*M	[6]
9	Fluorometric method	0.1–10 *μ*M	0.01 *μ*M	This work

**Table 2:** Detection of ATP in bovine serum using the fluorescence sensing platform.

Sample	Found amount (*μ*mol·L^−1^)	Added amount (*μ*mol·L^−1^)	Detection amount (*μ*mol·L^−1^)	Recovery (%)	Relative standard deviation (%, *n* = 3)
Bovine serum	0.1	0.1	0.197	98.5	2.6
		0.2	0.2874	95.8	1.9
	1	1	2.072	103.6	2.3
		2	2.919	97.3	1.0

## Data Availability

The data used to support the findings of this study are available from the corresponding author upon request.
